# Mathematical modelling of the automated FADU assay for the quantification of DNA strand breaks and their repair in human peripheral mononuclear blood cells

**DOI:** 10.1186/s13628-014-0009-z

**Published:** 2014-09-09

**Authors:** Michael Junk, Judy Salzwedel, Thilo Sindlinger, Alexander Bürkle, Maria Moreno-Villanueva

**Affiliations:** 1 Numerics group, Department of Mathematics and Statistics, Universität Konstanz, Konstanz, D-78457, Germany; 2 Molecular Toxicology Group, Department of Biology, University of Konstanz, Konstanz, D-78457, Germany

**Keywords:** FADU, DNA repair, DNA strand breaks, Mathematical model

## Abstract

**Background:**

Cells continuously undergo DNA damage from exogenous agents like irradiation or genotoxic chemicals or from endogenous radicals produced by normal cellular metabolic activities. DNA strand breaks are one of the most common genotoxic lesions and they can also arise as intermediates of DNA repair activity. Unrepaired DNA damage can lead to genomic instability, which can massively compromise the health status of organisms. Therefore it is important to measure and quantify DNA damage and its repair.

**Results:**

We have previously published an automated method for measuring DNA strand breaks based on fluorimetric detection of alkaline DNA unwinding [1], and here we present a mathematical model of the FADU assay, which enables to an analytic expression for the relation between measured fluorescence and the number of strand breaks.

**Conclusions:**

Assessment of the formation and also the repair of DNA strand breaks is a crucial functional parameter to investigate genotoxicity in living cells. A reliable and convenient method to quantify DNA strand breakage is therefore of significant importance for a wide variety of scientific fields, e.g. toxicology, pharmacology, epidemiology and medical sciences.

## Background

The fact that the DNA denatures (¿unwinds¿) under alkaline conditions has been used to establish FADU assay for measuring DNA strand breaks [2]. This method is based on the limited denaturation of the DNA under precisely controlled conditions of pH, temperature and time. Under these conditions only chromosome ends plus ¿open sites¿ in the DNA serve as starting points for the unwinding, which proceeds bidirectionally from internal damage sites. After stopping the unwinding process at a specified time, a dye is added that interacts with the remaining double stranded DNA and during contact with DNA emits a fluorescence signal whose intensity is inversely related to the number of DNA strand breaks originally present: the less fluorescence signal, the more DNA strand breaks.

When X-radiation is applied to living cells, the number of strand breaks is known to increase linearly with the dose applied [3],[4]. However, the fluorescence signal intensity depends on the dose in a nonlinear fashion and displays saturation. Previously, the percentage of double stranded DNA, remaining after the alkaline unwinding, relative to undenatured control or more precisely the decadic logarithm of the intensity ratio with and without unwinding has been used for that purpose, but this does not fully result in a linear function with radiation dose. In this article, we present a mathematical model that describes the effect of DNA unwinding on the resulting strength of the fluorescence signal and thereby captures the non-linear relationship more precisely.

The main assumptions of the model are large cell numbers, homogeneously distributed damage sites, and small variation of cell parameters like initial damage, susceptibility to applied damage, and potential unwinding distance from the site of damage. The resulting formula relates the relative fluorescence intensity to the applied X-ray dose and fits well with experiments. To obtain this result, it suffices to use elementary arguments from probability theory. In particular, a detailed biochemical model of the unwinding process as in [5]¿[8] is not required.

## Results

We consider *N* cells of identical type, which are subjected to a controlled DNA damage by a specified dose of X-radiation. After the treatment, the DNA in cell *i* exhibits a certain number of (single or double) strand breaks. We denote this damage *D*_*i*_ and split it into a pre-existing, initial contribution $D_{i}^{0}$ and an induced damage $D_{i}^{x}={?}_{i}d$ which is proportional to the applied dose *d* . In mathematical terms, the FADU assay assigns to each *D*_*i*_ a corresponding fluorescence signal *F*_*i*_ of the cell and the total fluorescence $F={?}_{i=1}^{N}F_{i}$ of all cells is measured. Ideally, the fluorescence *F*_*i*_ is related, after completion of the FADU assay, to the length of single and double stranded DNA in cell *i* according to

(1) $$F_{i}=c\left(B\left(1?L_{i}\right)+L_{i}\right)\text{.}$$

Here *L*_*i*_ is the relative length of double stranded DNA in the cell so that 1¿*L*_*i*_ is the relative length of DNA, which has been unwound. In particular, *c* is the fluorescence signal that is obtained in the case of no unwinding *L*_*i*_?=?1, and *cB* the one after complete unwinding *L*_*i*_?=?0 (we call *B* the relative background fluorescence). Normalizing the total fluorescence *F* with the one in the case of no unwinding *F*^0^? *=?Nc*, we obtain

(2) $$I=\frac{F}{F^{0}}=\frac{1}{N}\overset{N}{\underset{i=1}{?}}B\left(1?L_{i}\right)+L_{i}=B+\left(1?B\right)\frac{1}{N}\overset{N}{\underset{i=1}{?}}L_{i}$$

In other words, the relative fluorescence intensity *I* depends on the average relative length of double stranded DNA and the relative background fluorescence *B* under total unwinding. Later, the fact that *N* is large allows us to apply the mathematical *law of large numbers* to safely replace the average by an expected value.

It remains to model the relative length *L*_*i*_ of double stranded DNA after the unwinding process and its relation to the damage *D*_*i*_. Since we assume that the process happens identically and independently in each cell, we consider a generic situation and drop the cell index *i* in the following discussion.

Our basic assumption is that the break points *x*_*k*_ are uniformly distributed along the DNA strands and that the DNA unwinds a distance *?* to the left and to the right of *x*_*k*_ unless there is interference with the unwinding process going on at a neighboring break.

Since unwinding also starts at the ends of the chromosomes, it is useful to unify the description by hypothetically stringing the various chromosomes together. As far as unwinding is concerned, this does not change the result as long as the points which mark the relative stringing positions are added to the list of break points. This trick leads to an elegant description of the unwinding process: We start from *m* internal strand breaking points 0?<?z_1_?<???????<?z_*m*_?<?1 and associate to each *z*_*k*_ the two neighboring points $z_{k}^{\pm }=z_{k}\pm ?$ that mark the potential unwinding interval $\left[z_{k}^{?},z_{k}^{+}\right]$ which is also the actual unwinding if there is no interference with neighboring intervals. Obviously, such an interference appears between *z*_*k*_ and *z*_*k+*1_ if and only if $z_{k}^{+}>z_{k+1}^{?}$. Altogether, the contribution to the relative length *L* of double stranded DNA between *z*_*k*_ and *z*_*k+*1_ is ${\left(z_{k+1}^{?}?z_{k}^{+}\right)}_{+}$ where *u*_+_?=?max(*u*,?0) denotes the positive part of a number *u*. Introducing the auxiliary points *z*_0_?=?0, *z*_*m*?+?1_?=?1 and summing up all the contributions, we obtain

(3) $$L=\overset{m}{\underset{k=0}{?}}{\left(z_{k+1}^{?}?z_{k}^{+}\right)}_{+}=\overset{m}{\underset{k=0}{?}}{\left(z_{k+1}?z_{k}?2?\right)}_{+}=\overset{m}{\underset{k=0}{?}}{\left({?}_{k}?2?\right)}_{+}\text{,}$$

where *?*_*k*_?=?*z*_*k*?+?1_???*z*_*k*_ is the distance between consecutive break points. For *m* we use the splitting *m*?+?1?=?*D*^0^?+?*D*^*x*^ into the number of breaks *D*^*x*^?=?*?d* due to X-ray damage and the number *D*^0^ accounting for breaks at zero damage, resulting from normal cell metabolism. Note that in our model, *D*^0^ is at least the number of chromosomes because we have added their terminal points as artificial breaks. Due to the stochastic nature of the damaging process, the cell metabolism and the unwinding, the number of strand breaks per dose *?*, the zero dose value *D*^0^ and the unwinding distance *?* should be considered variables whose value may vary randomly from cell to cell.

To estimate the resulting distribution of the relative fluorescence signal ¿ with some manageable expression it seems worthwhile to simplify the model further, even if it requires additional assumptions. For example, the situation simplifies a lot if we assume that *all z*_1_,?¿,?*z*_*m*_ are independently and uniformly distributed in the interval [0,1], which means that the particular role of the original terminal chromosome points is dropped. Since in a normal diploid human cell, the number of chromosomes is only 46, this may be acceptable as long as *m* is comparably large.

According to [9], the uniform distribution of the points *z*_*k*_ implies that the distances *?*_*k*_?=?*z*_*k*?+?1_???*z*_*k*_, *k*?=?0,?¿,?*m* between the points are independent and identically distributed like ${?}^{\left(m\right)}=1?\sqrt[{m}]{U}$ where *U* is uniformly distributed on [0,1]. In other words, *?*^(*m*)^ has a probability density *m*(1???*x*)^*m*???1^ for *x*???[0,?1]. This result allows us to compute the conditional expectation

(4) $$E\left(L\left|m\right)\right)=\overset{m}{\underset{k=0}{?}}E{\left({?}_{k}?2?\right)}_{+}=\;\left(m+1\right)E{\left({?}^{\left(m\right)}?2?\right)}_{+}\text{.}$$

For a given value *?* we have in the case 2*?*?<?1

(5) $$E{\left({?}^{\left(m\right)}?2?\right)}_{+}=\overset{1}{\underset{2?}{?}}(x?2?)m{\left(1?x\right)}^{m?1}\mathrm{dx}=\frac{{\left(1?2?\right)}^{m+1}}{m+1}\text{,}$$

while *E*(*?*^(*m*)^???2*?*)_+_?=?0 when 2*?*???1. Combining this result with the previous formula, we find the conditional expectation

(6) $$E\left(L\left|D^{0},\right)\;?,\;?\right)={\left(1?2?\right)}_{+}^{D^{0}+\mathrm{?d}}\text{.}$$

In order to compute the unconditional expectation *E*(*L*), reasonable assumptions on the probability distributions of *D*^0^, *?* and *?* are required. Finally, *E*(*L*) depends in a complicated way on the dose *d*, the mean values $\bar{D^{0}},\bar{?},\bar{?}$, the corresponding variances and maybe other parameters of the distributions.

However, since little information on the distributions of *D*^0^, *?* and *?* is available, we continue in a different way. We assume that the unconditional expectation *E*(*L*) can be approximated with an expression of the form

(7) $$E\left(L\right)?{\left(1?2{?}_{\mathrm{eff}}\right)}^{D_{\mathrm{eff}}^{0}+{?}_{\mathrm{eff}}d}$$

which is a reasonable assumption when *D*^0^, *?* and *?* have negligible variance among all cells in the assay. Then, the effective values $D_{\mathrm{eff}}^{0},{?}_{\mathrm{eff}}$ and *?*_*eff*_ are close to the mean values $\bar{D^{0}},\bar{?},\bar{?}$.

Replacing the average in the formula for relative intensity *I* by the approximate expectation, we obtain

(8) $$I?P\left(d\right)=B+\left(1?B\right){\left(1?2{?}_{\mathrm{eff}}\right)}^{D_{\mathrm{eff}}^{0}+{?}_{\mathrm{eff}}d}\text{.}$$

More compactly, this relationship can be written in the form

(9) $$P\left(d\right)=B+\left(P_{0}?B\right)e^{?\mathrm{?d}}\text{,}$$

where the relation between the fit parameters *P*_0_,?*?* and the model parameters is

(10) $$P_{0}=B+\left(1?B\right){\left(1?2{?}_{\mathrm{eff}}\right)}^{D_{\mathrm{eff}}^{0}},\;?=?{?}_{\mathrm{eff}}\ln\left(1?2{?}_{\mathrm{eff}}\right)\text{.}$$

Once *B*,?*P*_0_,?*?* are determined, the equivalent dose value *d* corresponding to the relative intensity *P* can easily be determined by inversion

(11) $$d=?\frac{1}{?}\ln\frac{P?B}{P_{0}?B}\text{.}$$

Unfortunately, an estimate of $D_{\mathrm{eff}}^{0}+{?}_{\mathrm{eff}}d$ which is essentially the total number of strand breaks is not available if only *B*,?*P*_0_,?*?* are known. However, a related dose-equivalent value

(12) $$D_{\mathrm{tot}}=\frac{D_{\mathrm{eff}}^{0}+{?}_{\mathrm{eff}}d}{{?}_{\mathrm{eff}}}$$

is accessible. In fact, applying the logarithm to (*P*_0_???*B*)/(1???*B*) and using the relation for *?*, we find

(13) $$\frac{D_{\mathrm{eff}}^{0}}{{?}_{\mathrm{eff}}}=?\frac{1}{?}\;\ln\frac{P_{0}?B}{1?B}\text{.}$$

Together with the expression for the dose value *d*, we obtain a formula that relates *P* to the value *D*_*tot*_.

(14) $$D_{\mathrm{tot}}\left(P\right)=\frac{D_{\mathrm{eff}}^{0}+{?}_{\mathrm{eff}}d}{{?}_{\mathrm{eff}}}=?\frac{1}{?}\;\ln\frac{P?B}{1?B}$$

In order to test our model, we use experimental results from the experiment specified in *Material and Methods*. Here, three replicates of the relative intensities at seven dose values are available for eleven donors. We denote them *P*_*jkl*_ where the index *j*?=?0,?¿,?6 labels the X-ray dose, *k*?=?1,?¿,?11 represents the donor and *l*?=?1,?2,?3 indicates the independent repetitions of the experiment.

While each donor may exhibit different parameters *P*_0*k*_,?*?*_*k*_, the background intensity *B* can be assumed identical in all experiments. To determine the unknown coefficients, we want to minimize the objective function

(15) $$\overset{11}{\underset{k=1}{?}}\overset{6}{\underset{j=0}{?}}\overset{3}{\underset{l=1}{?}}{\left(B+\left(P_{0k}?B\right)\exp\left(?{?}_{k}d_{j}\right)?P_{\mathrm{jkl}}\right)}^{2}\underset{B,{?}_{k},P_{0k}}{?}\min$$

which leads to the same result as the minimization of

(16) $$z\left(B,?,P_{0}\right)=\overset{11}{\underset{k=1}{?}}\overset{6}{\underset{j=0}{?}}{\left(B+\left(P_{0k}?B\right)\exp\left(?{?}_{k}d_{j}\right)?{\bar{P}}_{\mathrm{jk}}\right)}^{2}$$

based on the averages

(17) $${\bar{P}}_{\mathrm{jk}}=\frac{1}{3}\overset{3}{\underset{l=1}{?}}P_{\mathrm{jkl}}\text{.}$$

To quantify the quality of the fit, we check how much of the total sum of squares

(18) $$SS_{\mathrm{tot}}={\overset{11}{\underset{k=1}{?}}\overset{6}{\underset{j=0}{?}}\left({\bar{P}}_{\mathrm{jk}}?\bar{P}\right)}^{2},\;\bar{P}=\frac{1}{77}\overset{11}{\underset{k=1}{?}}\overset{6}{\underset{j=0}{?}}{\bar{P}}_{\mathrm{jk}}$$

is explained by the fitting which leads to an *R*^2^-value

(19) $$R^{2}=1?\frac{SS_{\mathrm{err}}}{SS_{\mathrm{tot}}}\text{,}$$

where $SS_{\mathrm{err}}=z\left(B^{*},{?}^{*},P_{0}^{*}\right)$ is the residual in case of the optimal parameters. The optimization is carried out with the Levenberg-Marquardt algorithm of the Matlab software package with a resulting value of *R*^2^?=?0.99.

Confidence intervals for the fit parameters are computed with the bootstrap method [10] exploiting the information contained in the threefold repetition of each experiment. For each index combination (*j*,?*k*,?*l*), *N*?=?10000 bootstrap samples $P_{\mathrm{jkl}}^{n}$ are generated by drawing intensities randomly from the values *P*_*jk*1_,?*P*_*jk*2_,?*P*_*jk*3_ with replacement. Then the computation of the optimal parameters is repeated for each *n*?=?1,?¿,?*N* and the resulting distribution is used to construct 95%-confidence intervals.

The resulting value for *B* is

(20) B=0.122±0.008.

**Figure 1:**
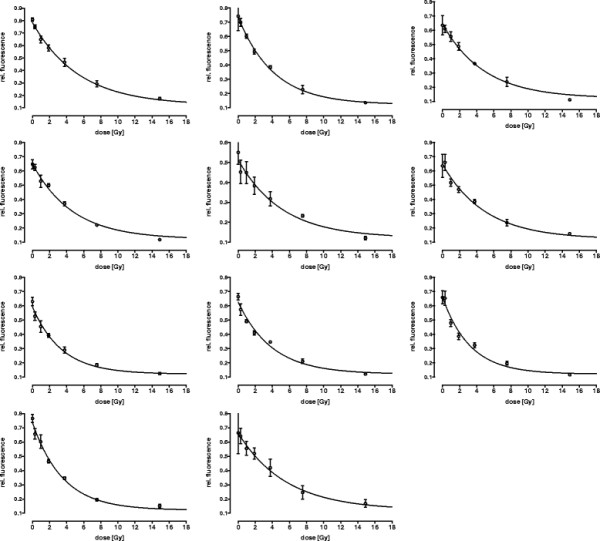
**Signal fluorescence depending on irradiation dose.** Human peripheral blood mononuclear cells (PBMC) were irradiated with several doses of x-ray. DNA strand breaks formation was measured using the automated FADU assay exactly as described [1].The Sybr-green fluorescence intensity, which is a direct marker of double-stranded DNA, decreases with increasing the irradiation dose, due to progressive alkaline unwinding of the DNA in the lysate starting from DNA ends or breaks. Circles represent the mean of three experimental replicates. Each graph represents the one donor.

The values *?*_*k*_,?*P*_0*k*_ for each donor are reported in Table 1 and the quality of the fit is demonstrated in Figure 1.

**Table 1:** Fit parameters for the relative fluorescence intensity

**Donor**	***P***_**0**_	***?***
1	0.79?±?0.01	0.18?±?0.02
2	0.74?±?0.06	0.24?±?0.04
3	0.64?±?0.04	0.21?±?0.03
4	0.65?±?0.02	0.21?±?0.02
5	0.51?±?0.04	0.20?±?0.05
6	0.65?±?0.05	0.20?±?0.04
7	0.60?±?0.03	0.30?±?0.04
8	0.63?±?0.02	0.25?±?0.02
9	0.66?±?0.04	0.31?±?0.05
10	0.74?±?0.03	0.28?±?0.02
11	0.66?±?0.08	0.18?±?0.06

The zero-dose value *P*_0_ shows a considerable variation among the donors ranging from 0.5 to 0.8, which is roughly three times the typical width of a confidence interval. In contrast, the exponential decay parameter has much less variation in the range 0.18 to 0.31, which amounts only to a single width of the typical confidence intervals.

## Discussion

DNA strand breaks are one of the most common genotoxic lesions. One of the methods used for measuring DNA strand breaks and their repair is the FADU assay. Under the selected alkaline conditions a large number of base pairs will be unwound to the right and left starting from each DNA ¿open side¿. Due to this amplification it is possible to detect very low number of DNA strand breaks. The main advantage of the FADU assay is that it can be performed in a fully automated fashion. However there are few methods that can be performed in a semi-automated version such as the COMET and ?H2AX assays. Even though these techniques provide useful tools for measuring DNA strand breaks in a high-throughput fashion, they have some disadvantages. A comprehensive comparison between these assays has been published before [11]. Briefly, ?H2AX assay is considered a very sensitive method that specifically detects double DNA strand breaks however in some cases the presence of ?H2AX foci in the absence of DNA damage has been demonstrated. The comet assay protocol includes many different steps making difficult the development of a fully automated version. Furthermore these also affect both intra-assay variability and inter-assay reproducibility.

We have previously published a modified and automated version of the FADU assay [1],[11],[12]. Compared with the original FADU assay [2], the automated FADU assay shows comparable sensitivity, yet increased robustness and throughput, and decreased operator time. However a suitable mathematical model, which allows translating the fluorescence intensity in DNA damage increases accuracy. In order to test whether the model is capable of describing the experimentally observed dependence between applied damage and resulting fluorescence intensity, we have measured the DNA strand breaks in peripheral mononuclear blood cells of 11 individuals. It turns out that the model is able to fit the dose-effect relation very accurately.

As shown in Table 1, the resulting model parameters *?* and *P*_0_, which describe the cell¿s susceptibility to DNA damage and the level of endogenous DNA strand breaks respectively, show a certain variation among the individuals which is stronger for the *P*_0_ value. An individual endogenous level of DNA strand breaks is expected; in general the importance of endogenous DNA damage has been reported before [13]. For example, it is known that DNA double-strand breaks (DSBs) accumulate in senescing human cell cultures and in ageing mice [14]. This is in accordance with our recent data showing that senescing human T lymphocytes have acquired more DNA strand breaks *in vivo* and an impaired DNA repair capacity *in vitro*[15]. Additionally, endogenous DNA strand breaks accumulation in human cells has been associated with nutrition [16], cancer [17], diabetes [18],[19], Down¿s syndrome [20], rheumatoid arthritis [21] and psychological stress disorders [22], among others. In other words, differences in the level of endogenous DNA strand breaks might reflect aging processes or health status of individuals.

The susceptibility to DNA damage *?* also shows variation between individuals. This finding is not surprising and has been described before. Odarigi and colleagues reported an inter-individual variance in chromosomal damage after x-irradiation [23]. Since radiation induces free oxygen radical formation, an individual damage response to x-ray might result from the different capacity of cells to scavenge these free radicals.

The investigation of DNA strand breaks and their repair is of great interest to toxicology, pharmacology, epidemiology and medical sciences at large. In general impaired DNA repair can lead to embryonic lethality, impaired growth, accelerated aging, shortened life span, and increased incidence of a variety of diseases, including a pronounced manifestation of cancer [24]¿[28]. Therefore robust methods for quantification of DNA damage and its repair are necessary.

Finally, in our model, induced DNA strand breaks are assumed to be uniformly distributed. However chromatin state could have an impact on the distribution of DNA strand breaks. Chromatin state differs depending on cell type and cell status (cell cycle phase and/or transcription) therefore it is important to take these aspects into consideration. A mathematical model of DNA unwinding considering the chromatin state would be an interesting task for future research, especially when comparing different cell types or cell status. A more refined model could be based on a probability distribution, which describes the susceptibility to breaking under X-ray exposure along the DNA strand.

## Conclusions

We have applied the above mathematical model for the quantification of DNA strand breaks in irradiated human PBMCs. This model can be also applied to other cell types and other DNA damaging agents as long as the damaging agent leads to uniformly distributed DNA strand breaks. Furthermore measuring the residual DNA strand breaks in damaged cells after several periods of time allow us to calculate the rate of repaired DNA strand breaks. Last but not least the amount of endogenous DNA strand breaks provides important information about the individual ¿*cell-damage-status*¿ and fluorescence signal after induced damage serves as biomarker of cell sensitivity to DNA strand breaks inducers, therefore an accurate model for DNA breakage quantification could be used for diagnostic and therapy purposes towards a personalized medicine, e.g. in cancer therapy.

## Methods

Selection of volunteers was carried out in accordance with the Declaration of Helsinki and ethical approval was obtained from the Ethics Committee of University of Constance. A signed Informed Consent was obtained from each subject. Blood from healthy volunteers aged 23¿55 was collected in sodium citrat tubes (Sarstedt, Nümbrecht, Germany). The automated FADU assay has previously been described in detail [1]. Briefly, peripheral blood mononuclear cells (PBMC) were isolated by Biocoll (Biochrom, Berlin, Germany) density gradient centrifugation. Cells were counted using a cell counting device (Casy® counter), pelleted (10 min, 200?*g*), and resuspended in isotonic buffer (0.25 M *meso*-inositol; 10 mM sodium phosphate, pH 7.4; 1 mM magnesium chloride) or RPMI-1640 medium (Invitrogen) containing 100 U/ml penicillin (Invitrogen), 100 mg/ml streptomycin (Invitrogen) and 10% fetal calf serum (FCS) at 4?×?10^6^ cells per ml. In order to induce DNA strand breaks several aliquots of 100 ?l cell suspension were irradiated on ice at a dose rate of 1.9 Gy/min using an X-ray generator (CHF Müller, Hamburg, Germany, 70 keV, 1 mm Al-filter). Qualitative behaviour has been modelled, but it still depends on few quantitative parameters, which where determined based on the results obtained from unwinding experiments after various (0.3, 1, 1.9, 3.8, 7.5 and 14.9 Gy) irradiation doses (Figure 1).

The detection of DNA strand breaks is based on progressive DNA unwinding (denaturation) under highly controlled conditions of alkaline pH, time and temperature. The starting points for the unwinding process are DNA ¿open sites¿ like replication forks or chromosome ends, but also DNA strand breaks. For monitoring DNA damage a commercially available fluorescent dye (SYBR Green®) is used as marker for double stranded DNA, and a decrease in the fluorescence intensity of SYBR Green® indicates an increase of DNA unwinding and consequently a greater number of DNA strand breaks.

T and P_0_ are controls to be run in parallel with the experimentally treated cells. In P0 samples alkaline unwinding is allowed and represents the DNA strand breaks under physiological conditions (*i.e.* without exogenous DNA damage). In T samples the neutralisation follows the lysis and therefore unwinding cannot take place; T-samples provide a measure of total DNA content and yield a fluorescence signal defined as 100%. P_x_ samples (P_1_,P_2_,P_3_,P_4_....P_x_) are the different extent of damage to be measured.

## Competing interests

The authors declare that they have no competing interests.

## Authors¿ contributions

The work presented here was carried out in collaboration between all authors. MMV defined the research theme and designed methods and experiments. JS carried out the laboratory experiments and analyzed the data. TS co-worked on data interpretation and discussed analyses. MJ developed the mathematical model and carried out the parameter fitting. AB co-designed experiments, discussed analyses, interpretation, and presentation. MMV and MJ wrote the paper. All authors have contributed to, seen and approved the manuscript.
